# Day-case robotic adrenalectomy under ERAS protocol: early outcomes from a single-centre experience

**DOI:** 10.1007/s00345-026-06465-3

**Published:** 2026-05-14

**Authors:** Alberto Costa Silva, Aishwarya Kaur, Safwaan Adam, Jan Ho, Awanish Shukla, Aziz Gulamhusein

**Affiliations:** 1 The Christie NHS Foundation Trust, Manchester, UK; 2 University Hospital Centre of São João, Faculty of Medicine, University of Porto, RISE – Health Research Network, Alameda Professor Hernâni Monteiro, Porto, 4200-319 Portugal

**Keywords:** Adrenalectomy, Enhanced recovery after surgery, Robotic surgical procedures, Ambulatory surgical procedures, Patient satisfaction

## Abstract

**Purpose:**

To evaluate the feasibility and outcomes of a day-case robotic adrenalectomy programme implemented within a structured Enhanced Recovery After Surgery (ERAS) framework.

**Materials and methods:**

A retrospective analysis was conducted of all patients who underwent day-case robotic adrenalectomy between November 2023 and March 2025. Procedures were performed via a transperitoneal approach using the da Vinci Xi^®^ system and managed under an ERAS protocol incorporating multimodal analgesia, early mobilisation, and omission of postoperative drains and catheters. A transversus abdominis plane (TAP) block was administered intraoperatively for analgesia. Outcomes included operative metrics, perioperative complications, 30-day readmissions, and patient-reported satisfaction measured by the Evaluation du Vécu de l’Anesthésie Générale (EVAN-G) questionnaire.

**Results:**

Thirteen patients were included. The median age was 49.0 years (46.0–60.0), and the median BMI was 30.0 kg/m² (28.0–36.5). Most patients were male (61.5%, *n* = 8), all were ASA grade 2, and 69.2% (*n* = 9) had an ECOG performance status of 0. The median estimated blood loss was 10.0 mL (5.0–10.0) and the median operative time was 56.0 min (30.5–75.0 min). A history of prior abdominal surgery was present in 38.5% of patients (*n* = 5). Indications included primary hyperaldosteronism (69.2%, *n* = 9), androgen-producing tumour (*n* = 1), RCC metastasis (*n* = 1), and adrenocortical carcinoma (*n* = 2). No intraoperative or postoperative complications occurred, no patients required readmission, and the median EVAN-G score was 94 (91–96). On preoperative imaging, the median tumour size was 16.0 mm (13.0–26.5 mm). Postoperative pathological analysis reported a median adrenal gland size of 63.0 mm (50.0–72.5 mm) and a median lesion size of 15.0 mm (9.0–22.5 mm).

**Conclusion:**

Day-case robotic adrenalectomy performed under an ERAS protocol is safe and well-tolerated in selected patients.

## Introduction

Minimally invasive adrenal surgery has gained widespread adoption following the introduction of laparoscopic and robotic adrenalectomy [1–3]. Day-case surgery is well established across a wide range of urological procedures offering advantages for both patients and healthcare systems [4]. Despite receiving renewed attention, robotic day-case approaches in adrenal surgery remains limited, primarily due to the technical complexity of adrenalectomy and the specific perioperative requirements related to anaesthetic and endocrine management [5, 6]. Therefore, current data on robotic day-case adrenalectomy are still scarce.

While the implementation of structured perioperative protocols—such as those guided by Enhanced Recovery After Surgery (ERAS) principles—can be particularly challenging in adrenal surgery, their integration has the potential to significantly improve patient outcomes and streamline perioperative care [6–9].

Our department is a high-volume robotic oncology centre with expertise in complex upper urinary tract procedures. Recently, we implemented a dedicated day-case robotic surgery programme.

This study aimed to evaluate the feasibility, safety, and patient-reported outcomes following day-case robotic adrenalectomy performed under an ERAS protocol.

## Materials and methods

This is a single-centre, prospective cohort study between January 2024 and March 2025. Institutional Review Board approval was obtained, and all patients provided informed consent in accordance with the Declaration of Helsinki. The study included all consecutive patients who underwent day-case robotic adrenalectomy who were managed through a predefined ERAS protocol.

The following variables were collected from the patient records: demographic data (age, sex, BMI, smoking status, diabetes), preoperative clinical status (ASA grade, ECOG performance status, endocrinological diagnosis, previous abdominal surgery), tumour characteristics (side, size on imaging, pathology-confirmed lesion size and adrenal size), operative details (surgical time – includes docking and console time -, estimated blood loss, surgical indication), and logistical factors (home-to-hospital distance). Postoperative outcomes included intraoperative and postoperative complications, need for hospital contact, readmissions, and evidence of adrenal insufficiency.

Patients were selected for day-case robotic adrenalectomy based on predefined clinical criteria, including an American Society of Anesthesiologists (ASA) physical status of 2 or less and an Eastern Cooperative Oncology Group (ECOG) performance status of 0 to 1, absence of phaeochromocytoma, and adequate social support at home to safely facilitate same-day discharge.

All patients underwent comprehensive preoperative evaluation. This included endocrinological assessment to determine hormonal activity and optimise adrenal-related conditions, an anaesthetic evaluation to assess suitability for day-case surgery, and a urological consultation for surgical planning. A multidisciplinary team made the final decision regarding surgical eligibility. Patients received detailed preoperative counselling covering the surgical technique, expectations for the day-case pathway, and recovery process.

All surgeries were performed by a single urologist assisted by a fellow using a robotic approach with Da Vinci Xi^®^ Surgical System (Intuitive Surgical Inc., Sunnyvale, CA, USA). Antibiotic prophylaxis was administered using amoxicillin and clavulanic acid 1.2 g (or Metronidazole 500 mg for penicillin-allergic patients) plus Gentamicin 1.5 mg/kg, within 30 min pre-incision.

**Fig. 1:**
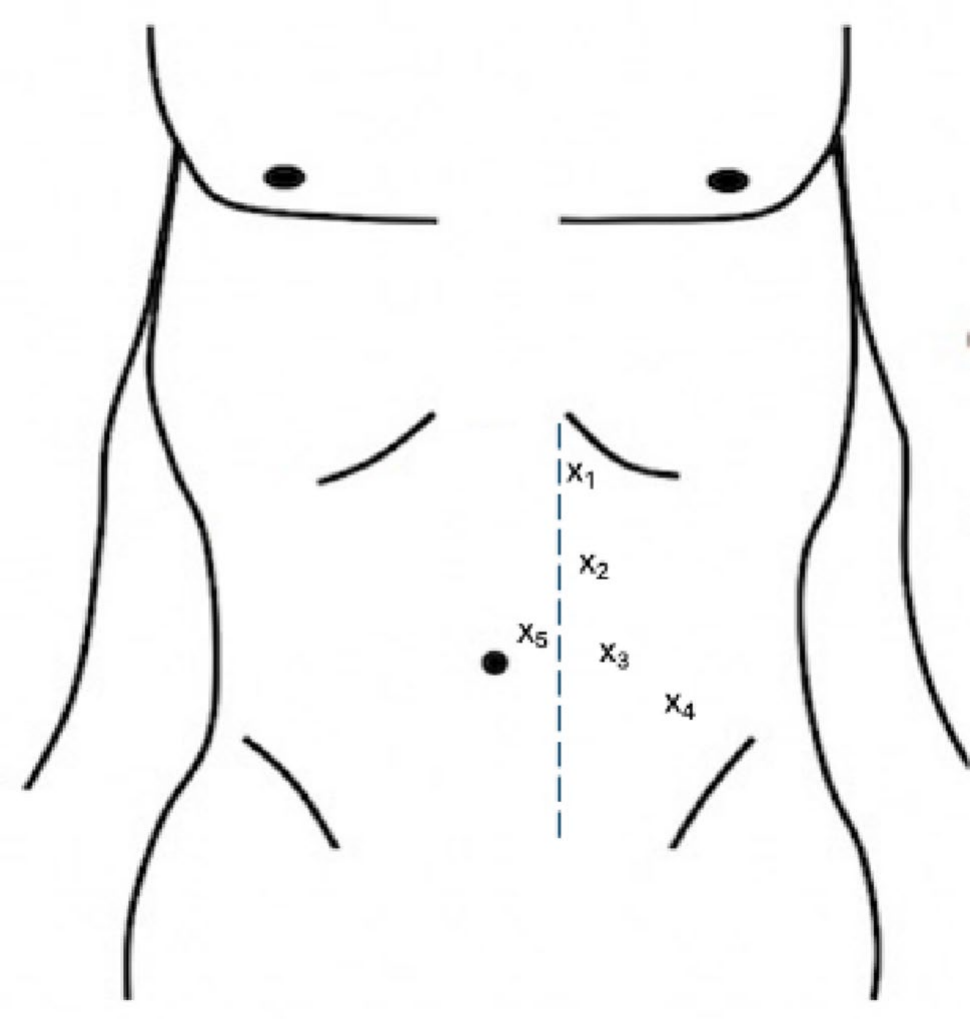
Trocar placement for left robotic adrenalectomy. X_1_ – 8 mm, Fenestrated bipolar forceps; X_2_ – 8 mm, Camera: X_3_ – 8 mm, Monopolar curved scissors; X_4_ – 8 mm, ProGrasp forceps; X_5_ − 12 mm, Airseal^®^; Trocar placement for right-sided procedure was done in a mirror fashion.

A Foley catheter was inserted at the start and removed at the end of the operation. The patient was placed in the flank position, and a total of five ports were used—four robotic (camera, ProGrasp forceps, monopolar curved scissors, and fenestrated bipolar forceps) and one assistant port (AirSeal^®^) (Fig. 1). A transperitoneal approach was performed with pneumoperitoneum established at a pressure of 8 mmHg. Dissection was performed using bipolar and monopolar energy devices with minimal bowel mobilisation. The adrenal vein was secured using Hem-o-Lok™ clips and the specimen was retrieved in a protected extraction bag which was removed by the AirSeal^®^ Port. A transversus abdominis plane (TAP) block was administered under direct vision with maximum dose of 2 mg/kg 0.5% Bupivacaine at the end of the procedure, followed by a further 20 ml injected into port site and a 50 mg diclofenac suppository was administered before extubating. No surgical drains or urinary catheters were left postoperatively.

A standardized ERAS protocol was followed. Patients were allowed clear fluids up to two hours and solid food up to six hours prior to surgery. There was no routine use of nasogastric tube or bowel preparation. Intraoperative management focused on avoiding hypothermia using active warming with an air blanket and maintaining fluid balance. All patients were extubated in the operating room and monitored in the post-anaesthesia care unit for one hour before being transferred to the ward. Oral intake and ambulation were resumed two hours after surgery. Pain was managed using a multimodal regimen that included a TAP block, diclofenac suppository, regular paracetamol 1 g orally four times daily for 7 days, ibuprofen 400 mg orally three times daily for 3 days, with omeprazole 20 mg orally once a day, and codeine phosphate 30 mg orally four times daily for 7 days as required.

Discharge was considered appropriate when the patient had stable vital signs, effective pain control on oral medication, independent mobility, and could tolerate food and fluids and had passed urine. Endocrinology and Urology reviews were completed prior to discharge, with a follow-up endocrinology assessment conducted within one week after surgery. All patients were provided with direct telephone access to the hospital nursing team for any concerns at home. Additionally, patients were followed up with two telephone calls within 24 and 72 h of discharge which were completed by the ERAS Team. A urologic follow-up was scheduled six weeks postoperatively, following a multidisciplinary discussion of the histopathological results.

Postoperative patient satisfaction was assessed using the Evaluation du Vecu de l’Anesthesie Generale (EVAN-G) questionnaire [10, 11].

### Statistical analysis

The data were collected and analysed using the Statistical Package for the Social Sciences (version 27; IBM, Chicago, IL, USA). The Kolmogorov–Smirnov test was used to evaluate the distribution of the parameters. Normally distributed continuous variables are presented as the mean ± standard deviation, while non-normally distributed continuous variables are presented as the median (interquartile range-IQR).

## Results

A total of thirteen patients were submitted to day-case robotic adrenalectomy. The median age was 49.0 years (46.6–60.0), and the median body mass index (BMI) was 30.0 kg/m² (28.0–36.5). Most patients were male (61.5%, *n* = 8). All patients were classified as ASA grade 2, and 69.2% (*n* = 9) had ECOG performance status of 0. Tumour laterality was predominantly right sided, observed in 61.5% (*n* = 8). On preoperative imaging, the median tumour size was 16.0 mm (13.0–26.5 mm). Postoperative pathological analysis reported a median adrenal gland size of 63.0 mm (50.0–72.5 mm) and a median lesion size of 15.0 mm (9.0–22.5 mm). A history of previous abdominal surgery was present in 38.5% (*n* = 5) and the median patient home-hospital distance was 10.0 (4.6–19.3) miles. The median estimated intraoperative blood loss was 10.0 mL (5.0–10.0 mL) and the median operative time was 56.0 min (30.5–75.0 min).

Surgical indications included primary hyperaldosteronism in most patients (69.2%, *n* = 9), with the remaining cases distributed among androgen-secreting adrenal tumours (*n* = 1), metastatic renal cell carcinoma (*n* = 1), and adrenocortical carcinoma (*n* = 2). Patient demographics and perioperative data are summarized in Table 1.

**Table 1:** Patient demographics and perioperative parameters for day-case robotic adrenalectomy

Case	Surgical time, minutes	Age, years	Gender	Home-hospital distance, miles	BMI, kg/m^2^	ASA	ECOG	Prior abdominal surgery	Smoker	Diabetes	Lesion side	Adrenal size, mm	Lesion size, mm	Blood loss, mL	Surgical indication
1	74	49	Male	13.4	38.0	2	0	No	No	No	Left	80.0	8.0	10	Primary hyperaldosteronism
2	74	49	Male	3.5	30.0	2	0	No	No	No	Left	70.0	13.0	40	Primary hyperaldosteronism
3	54	46	Male	19.6	34.0	2	0	No	Yes	No	Right	60.0	16.0	10	Primary hyperaldosteronism
4	27	48	Male	19.0	42.0	2	0	Appendectomy	Yes	No	Right	75.0	22.0	10	Primary hyperaldosteronism
5	34	62	Male	5.4	28.0	2	1	No	Yes	No	Right	70.0	13.0	5	Primary hyperaldosteronism
6	44	67	Male	10.0	29.0	2	1	Inguinal hernia	No	Yes	Left	75.0	14.0	5	Primary hyperaldosteronism
7	26	58	Female	4.0	23.0	2	0	No	Yes	No	Right	50.0	15.0	5	Androgen-producing tumour
8	27	40	Female	6.0	27.0	2	0	No	No	No	Right	50.0	7.0	5	Primary hyperaldosteronism
9	56	62	Male	16.0	28.0	2	1	Left nephrectomy	Yes	No	Right	45.0	23.0	5	RCC metastasis
10	77	47	Male	34.0	35.0	2	0	No	Yes	No	Left	53.0	16.0	10	Primary hyperaldosteronism
11	84	43	Female	20.0	35.0	2	0	No	Yes	No	Right	65.0	60.0	10	Adrenocortical carcinoma
12	60	56	Female	7.3	42.0	2	1	Caesarean section	No	No	Left	30.0	28.0	20	Adrenocortical carcinoma
13	66	53	Female	4.5	28.0	2	0	Hysterectomy	Yes	No	Right	63.0	7.0	5	Primary hyperaldosteronism

ASA – American Society of Anaesthesiologists score, ECOG – Eastern Cooperative Oncology Group, BMI – Body Mass Index, RCC – Renal Cell Carcinoma, mm – millimetres, mL – millilitres, kg/m² – kilograms per square meter

There were no intraoperative or postoperative complications recorded. No cases of postoperative adrenal insufficiency were observed. Two patients (15.4%) contacted the hospital directly via telephone for advice but did not require any further medical intervention. No unplanned readmissions or postoperative emergency visits were observed within the follow-up period. All patients planned for day-case robotic adrenalectomy were successfully discharged on the day of surgery, with no unplanned inpatient admissions, and no unplanned readmissions or postoperative emergency visits were observed within the follow-up period. The median global EVAN-G score was 94.0 **(**91.0–96.0).

## Discussion

In the context of day-case robotic adrenalectomy, two considerations must be addressed: the rationale for adopting a robotic approach and the justification for a same-day discharge model. Current literature provides comparative data on robotic versus laparoscopic adrenalectomy based on inpatient data. Three meta-analyses have reported that robotic adrenalectomy is associated with a slightly longer operative time (157–172 min vs. 150–162 min). Robotic adrenalectomy demonstrated benefits in terms of reduced hospital stay (2.38–3.06 days vs. 2.88–4 days) and lower intraoperative blood loss (45.7–66.9 mL vs. 74.8–85.3 mL). The rates of intraoperative complications were comparable between approaches (4.8% for robotic adrenalectomy vs. 3.9% for laparoscopic adrenalectomy). Robotic adrenalectomy was consistently associated with higher overall procedural costs (8695 USD for robotic adrenalectomy vs. 4560 for laparoscopic adrenalectomy); however, these figures reflect a private healthcare context in the United States [12–15]. Multiple studies have supported the feasibility of outpatient laparoscopic adrenalectomy, showing that same-day discharge can be achieved reliably without elevating the risk of postoperative complications or hospital readmissions [16, 17]. For robotic adrenalectomy in the inpatient setting, evidence indicates that perioperative outcomes are comparable to those of laparoscopy [15, 18, 19]. In one study, five patients underwent robotic adrenalectomy in a day-case setting. The median operative time was 120 min, with an estimated blood loss of 50 mL. There were no intraoperative or postoperative complications reported, and all patients were discharged the same day with no readmissions [20]. Similarly, in other report fifteen outpatient robotic adrenalectomies were performed with a mean operative time of 134 ± 10 min. The procedures were well tolerated, with one case of adrenal insufficiency [18]. Moreover, the introduction of single-port robotic platforms has further facilitated same-day discharge [15, 21].

During the study period, a total of 47 robotic adrenalectomies were performed. Of these, 13 selected patients underwent planned day-case robotic adrenalectomy. Our series demonstrated a median operative time of 56 min (with some procedures completed in less than 30 min), shorter than previous robotic or laparoscopic reports. Notably, this was achieved despite inclusion of surgical training components. Our cohort included patients with higher body mass index values, with a median BMI of 30.0 kg/m². Only one patient had a BMI below 25 kg/m², and the highest recorded BMI was 42 kg/m². Estimated blood loss was also minimal, with a median of 10 mL. Additionally, the median adrenal gland size was 63.0 mm, with a median tumour size of 16 mm, and there was a predominance of functional adrenal adenomas, particularly cases of primary hyperaldosteronism. Importantly, no intraoperative or postoperative complications occurred, and there were no cases of postoperative adrenal insufficiency or readmission, consistent with the favourable safety profile reported in prior outpatient robotic adrenalectomy series. Other contextual factors likely contributed to the success of the day-case approach. All patients resided in the vicinity of the hospital, facilitating rapid access to care if needed. Furthermore, a dedicated 24-hour phone line was provided, allowing direct communication with the hospital nursing team for any postoperative concerns. Patient selection was also critical: all individuals were deemed fit for day-case surgery based on favourable ASA (≤ 2) and ECOG (0–1) scores. Notably, a history of prior abdominal surgery—present in five patients—did not appear to impact operative times, which ranged from 27 to 66 min in these cases.

Robotic platforms are increasingly adopted worldwide and have become the standard approach for many urological procedures [22]. This trend is reflected in current practice, as conventional urologic laparoscopy is no longer routinely performed in our centre. One of the primary concerns surrounding robotic adrenalectomy is the associated cost [23, 24]. However, the additional expenses linked to the robotic platform may be offset by several factors, including reduced length of hospital stay, increased patient referrals due to procedural efficiency, and improved postoperative outcomes, particularly in complex cases [25]. In our institutional context, where access to robotic platforms is well-established and patients benefit from a streamlined fast-track pathway without compromising care quality, outpatient robotic adrenalectomy represents a viable and cost-conscious option. The increasing competition among surgical technology providers is helping to lower the costs traditionally associated with robotic surgery, thereby enhancing the economic viability of this approach [26, 27].

The inclusion of patient-reported outcome measures, specifically the EVAN-G questionnaire, provided a patient-centred dimension to the assessment of our day-case robotic adrenalectomy protocol. The EVAN-G is a validated tool that measures patient satisfaction across seven domains—attention, privacy, information, pain, discomfort, waiting time, and overall satisfaction—on a 0–100 scale [10, 11]. The uniformly high scores observed in our cohort reinforce the safety, comfort, and overall acceptability of same-day robotic adrenalectomy.

This initiative forms part of a comprehensive adrenal programme jointly established by the Urology and Endocrinology departments. The programme encompasses joint multidisciplinary clinics, a full range of endocrine diagnostic investigations (including adrenal vein sampling), and the management of complex adrenal cases through both open and robotic approaches. This integrated model enables coordinated preoperative assessment, optimised perioperative endocrine management, and structured postoperative follow-up.

This study has several limitations. First, the small sample size of thirteen patients limits the generalizability of the findings. The retrospective design introduces the possibility of selection and reporting biases, and the absence of a control group precludes direct comparisons. Additionally, all patients were highly selected, which may not reflect the broader population of individuals undergoing adrenal surgery. Importantly, patients with pheochromocytoma were excluded, as these cases require more complex perioperative haemodynamic monitoring and postoperative observation, making them less suitable for a day-case model. The single-centre setting and high level of surgical expertise further limit the external validity, as outcomes may not be replicable in lower-volume centres. In this series, the transperitoneal approach was adopted in all cases based on surgeon preference and institutional experience. The authors acknowledge, however, that the retroperitoneal approach is a valid alternative, offering potential advantages such as direct access to the adrenal gland and avoidance of intraperitoneal organ mobilisation [28]. Furthermore, the increasing adoption of single-port robotic platforms may expand the applicability of both transperitoneal and retroperitoneal approaches. Finally, while the study references potential economic benefits, no formal cost-effectiveness analysis was performed. These factors highlight the need for larger, prospective, multicentre studies to validate the feasibility, safety, and economic impact of day-case robotic adrenalectomy.

Day-case robotic adrenalectomy, when performed under ERAS protocols in high-volume centres, appears to be a safe and efficient for selected patients. Further prospective, multicentre studies incorporating patient-reported outcomes and economic analyses are needed to establish definitive clinical and cost-effectiveness of day-case robotic adrenalectomy.

## Data Availability

The data that support the findings of this study are restricted by local ethics comissions. The data are, however, available from the authors upon reasonable request and with the permission of ethics comitee.

## References

[1] Michel Gagner (1992) Laparoscopic adrenalectomy in Cushing’s syndrome and pheochromocytoma. N Engl J Med 327:1033 10.1056/NEJM199210013271417 1387700

[2] Horgan S, Vanuno D (2001) Robots in laparoscopic surgery. J Laparoendosc Adv Surg Tech. (11):415–419 10.1089/10926420152761950 11814134

[3] Hubens G, Ysebaert D, Vaneerdeweg W, Chapelle T, Eyskens E (1999) Laparoscopic adrenalectomy with the aid of the AESOP 2000 robot. Acta Chir Belg 99(3):125–127 10427347

[4] Azhar RA, Bochner B, Catto J, Goh AC, Kelly J, Patel HD et al (2016) Enhanced recovery after urological surgery: a contemporary systematic review of outcomes, key elements, and research needs. Eur Urol 70:176–187 10.1016/j.eururo.2016.02.051 PMC5514421 26970912

[5] Pellegrino A, Briganti A, Crivellaro S (2025) Same-day Outpatient Robotic Surgery in Urology. Eur Urol Focus 11:11–14 10.1016/j.euf.2025.03.006 40113515

[6] Rohi A, Olofsson MET, Jakobsson JG (2022) Ambulatory anesthesia and discharge: an update around guidelines and trends. Curr Opin Anaesthesiol 35(6):691–697 36194149 10.1097/ACO.0000000000001194

[7] van de Wiel ECJ, Mulder J, Hendriks A, Booij Liewes-Thelosen I, Zhu X, Groenewoud H et al (2024) Adrenal fast-track and enhanced recovery in retroperitoneoscopic surgery for primary aldosteronism improving patient outcome and efficiency. World J Urol 42(1):187 10.1007/s00345-024-04911-8 PMC10959772 38517537

[8] Lelli G, Micalizzi A, Iossa A, Fassari A, Concistre A, Circosta F et al (2024) Application of enhanced recovery after surgery (ERAS) protocols in adrenal surgery: a retrospective, preliminary analysis. J Minim Access Surg 20(2):163–168 37282440 10.4103/jmas.jmas_319_22 PMC11095811

[9] Yan Y, Cheng J, Chen K, Liu TF, Ning G (2022) Better clinical benefits and potential cost saving of an enhanced recovery pathways for laparoscopic adrenalectomy. Gland Surg 11(1):23–34 35242666 10.21037/gs-21-504 PMC8825523

[10] Auquier P, Pernoud N, Bruder N, Simeoni MC, Auffray JP, Colavolpe C et al (2005) Development and validation of a perioperative satisfaction questionnaire. Anesthesiology [Internet] 102:1116–1139 Available from: www.anesthesiology.org 15915023 10.1097/00000542-200506000-00010

[11] Maurice-Szamburski A, Bringuier S, Auquier P, Capdevila X (2024) From pain level to pain experience: redefining acute pain assessment to enhance understanding of chronic postsurgical pain. Br J Anaesth 133(5):1021–1027 39332996 10.1016/j.bja.2024.08.003

[12] Economopoulos KP, Mylonas KS, Stamou AA, Theocharidis V, Sergentanis TN, Psaltopoulou T et al (2017) Laparoscopic versus robotic adrenalectomy: a comprehensive meta-analysis. International Journal of Surgery, vol 38. Elsevier Ltd, pp 95–104 10.1016/j.ijsu.2016.12.118 28043926

[13] Esposito G, Mullineris B, Colli G, Curia S, Piccoli M (2025) Robotic versus laparoscopic adrenalectomy for adrenal tumors: an up-to-date meta-analysis on perioperative outcomes. Cancers. Multidisciplinary Digital Publishing Institute (MDPI) 10.3390/cancers17010150 PMC11719468 39796777

[14] Brandao LF, Autorino R, Laydner H, Haber GP, Ouzaid I, De Sio M et al (2014) Robotic versus laparoscopic adrenalectomy: a systematic review and meta-analysis, vol 65. Elsevier, European Urology, pp 1154–1161 10.1016/j.eururo.2013.09.021 24079955

[15] Abaza R, Murphy C, Bsatee A, Brown DH, Martinez O (2021) Single-port robotic surgery allows same-day discharge in majority of cases. Urology 148:159–165 33217457 10.1016/j.urology.2020.08.092

[16] Galata’ G, Alexandrou K, Talat N, Hanschell H, Al-Lawati A, Klang P et al (2023) Defining the feasibility of same day adrenalectomy - a prospective matched cohort study. Surg Open Sci 14:75–80 37519329 10.1016/j.sopen.2023.07.009 PMC10374961

[17] Shariq OA, Bews KA, McKenna NP, Dy BM, Lyden ML, Farley DR et al (2021) Is same-day discharge associated with increased 30-day postoperative complications and readmissions in patients undergoing laparoscopic adrenalectomy? Surgery (United States). Mosby Inc., pp 289–297 10.1016/j.surg.2020.08.018 33008614

[18] Moughnyeh M, Lindeman B, Porterfield JR, Dream S (2020) Outpatient robot-assisted adrenalectomy: Is it safe? Am J Surg 220:296–297 10.1016/j.amjsurg.2020.04.037 32402438

[19] Gartland RM, Fuentes E, Fazendin J, Fong ZV, Stephen A, Porterfield JR et al (2021) Safety of outpatient adrenalectomy across 3 minimally invasive approaches at 2 academic medical centers. Surg (United States) 169(1):145–149 10.1016/j.surg.2020.03.026 32409169

[20] Ragavan N, Bafna S, Thangarasu M, Prakash S, Paul R, Chirravur P et al (2021) Day-case robot-assisted laparoscopic surgery: feasibility and safety. Turk J Urol 47(1):30–34 33135995 10.5152/tud.2020.20414 PMC7815236

[21] Hegazy M, Toubasey S, Giudice F, Del, Challacombe B (2025) Contemporary practice in adrenal surgery: a review of multiport versus single-port and transperitoneal versus retroperitoneal approaches. European Urology Focus, vol 11. Elsevier B.V., pp 26–28 10.1016/j.euf.2025.05.004 40368722

[22] Pyrgidis N, Volz Y, Ebner B, Westhofen T, Staehler M, Chaloupka M et al (2025) Evolution of robotic urology in clinical practice from the beginning to now: results from the GRAND Study Register. Eur Urol Focus 11(1):109–117 39209568 10.1016/j.euf.2024.08.004

[23] De Crea C, Arcuri G, Pennestrì F, Paolantonio C, Bellantone R, Raffaelli M (2020) Robotic adrenalectomy: Evaluation of cost-effectiveness. Gland Surgery, vol 9. AME Publishing Company, pp 831–839 10.21037/gs.2020.03.44 PMC7347830 32775276

[24] Mrinal Pahwa (2015) Robot assisted adrenalectomy: a handy tool or glorified obsession? Gland Surg 4(4):279–282 26312212 10.3978/j.issn.2227-684X.2015.05.01 PMC4523626

[25] Mihai R, Donatini G, Vidal O, Brunaud L (2019) Volume-outcome correlation in adrenal surgery—an ESES consensus statement. Langenbeck’s Arch Surg 404:795–806 10.1007/s00423-019-01827-5 PMC6908553 31701230

[26] Landi S, Maistri G, Orsini LP, Leardini C, Malandra S, Antonelli A (2025) Supporting managerial decisions: a comparison of new robotic platforms through time-driven activity-based costing within a value-based healthcare framework. BMC Health Serv Res 25(1):470 10.1186/s12913-025-12598-9 PMC11954269 40158085

[27] Steffens D, McBride KE, Hirst N, Solomon MJ, Anderson T, Thanigasalam R et al (2023) Surgical outcomes and cost analysis of a multi-specialty robotic-assisted surgery caseload in the Australian public health system. J Robot Surg 17(5):2237–2245 37289337 10.1007/s11701-023-01643-6 PMC10492768

[28] Manyalich-Blasi M, Saavedra-Pérez D, Guzman LMF, Llompart MM, Brito JA, Espert JJ et al (2025) Robotic posterior retroperitoneoscopic adrenalectomy: initial experience with Hugo™ RAS system. J Robot Surg 19(1):298 10.1007/s11701-025-02414-1 PMC12170789 40523980

